# Signet-ring cell carcinoma of the duodenal bulb presenting with gastrointestinal hemorrhage: a case report and literature review

**DOI:** 10.1186/s12876-022-02267-0

**Published:** 2022-05-09

**Authors:** Nan Ye, Xiaoxiao Bao, Xiaokang Zhao, Bin Wang

**Affiliations:** 1 Present Address: Department of Hepatobiliary Surgery, Dongyang People’s Hospital, Zhejiang Province, Jinhua, 322100 China; 2 Present Address: Department of Pathology, Dongyang People’s Hospital, Zhejiang Province, Jinhua, 322100 China; 3 Present Address: Department of Gastrointestinal Surgery, Dongyang People’s Hospital, Zhejiang Province, Jinhua, 322100 China

**Keywords:** Duodenal, Bulb, Signet-ring cell carcinoma

## Abstract

**Background:**

Primary duodenal cancer (PDC) is rare, especially signet-ring cell carcinoma (SRCC) of the duodenal bulb, and it is commonly misdiagnosed as an ulceration. Here, we report a rare case of SRCC of the duodenal bulb presenting with gastrointestinal hemorrhage in an 82-year-old man.

**Case presentation:**

An 82-year-old man was admitted for gastrointestinal hemorrhage. Physical examination revealed upper abdominal tenderness and pale appearance, but was otherwise unrevealing. Laboratory workup was significant for anemia. Imaging showed no abnormalities. Two endoscopic evaluations along with interventional embolization were attempted and, unfortunately, adequate hemostasis was not achieved, resulting in distal subtotal gastrectomy, including the duodenal bulb. SRCC of the duodenal bulb was diagnosed based on pathology after surgery. Post-operatively, the patient experienced persistent gastrointestinal bleeding. Family declined further intervention and the patient eventually died one month post-resection.

**Conclusions:**

SRCC in the duodenal bulb is difficult to diagnose. For those with high-risk factors, endoscopic examination and biopsy are recommended. For patients who can receive radical tumor resection, pancreaticoduodenectomy (PD) is considered a first-line option. Early diagnosis and resection have been shown to improve prognosis.

## Background

Primary duodenal cancer (PDC) is rare and accounts for just 0.3–0.5% of gastrointestinal cancers [1]. Most PDCs are histopathologically adenocarcinomas, while signet-ring cell carcinoma (SRCC) is extremely rare, accounting for approximately 1% of duodenal adenocarcinomas [2]. It has been reported that over 96% of SRCC cases occur in the stomach. Although rare, other organs may be affected, including the breast, gallbladder, pancreas, urinary bladder and bowel [3]. Since Sekoguchi et al. first reported SRCC of the duodenal ampulla in 1979, few cases of duodenal SRCC have been reported [4]. To our knowledge, SRCC within the duodenal bulb is an even rarer entity with no more than 15 cases reported in the English literature to date [5]. Herein, we report a rare case of SRCC of the duodenal bulb presenting with gastrointestinal hemorrhage in an 82-year-old man.

## Case presentation

An 82-year-old male was admitted to the Department of Gastroenterology in November 2020 with intermittent melena for a duration of 2 months and hematemesis over the past 10 h. He had a history of hypertension, coronary artery disease after stenting, gout and diabetes mellitus, and he had been treated with clopidogrel hydrogen sulphate tablets since stenting. The patient was treated with analgesics that he could not describe before admission because of epigastric discomfort. His brother died of lung cancer. In 2014, he received endoscopic treatment due to a gastric ulcer complicated with hemorrhage in our hospital, and pathology revealed chronic active gastritis of the gastric mucosa. Two weeks before admission, the patient had a large amount of melena, and endoscopy was performed, which revealed multiple lesions of ulceration in the descending part of the duodenum. Gastroenterography showed the image of barium protruding out of the lumen in the descending part of the duodenum, which was considered a diverticulum. No biopsy was performed.

**Fig. 1:**
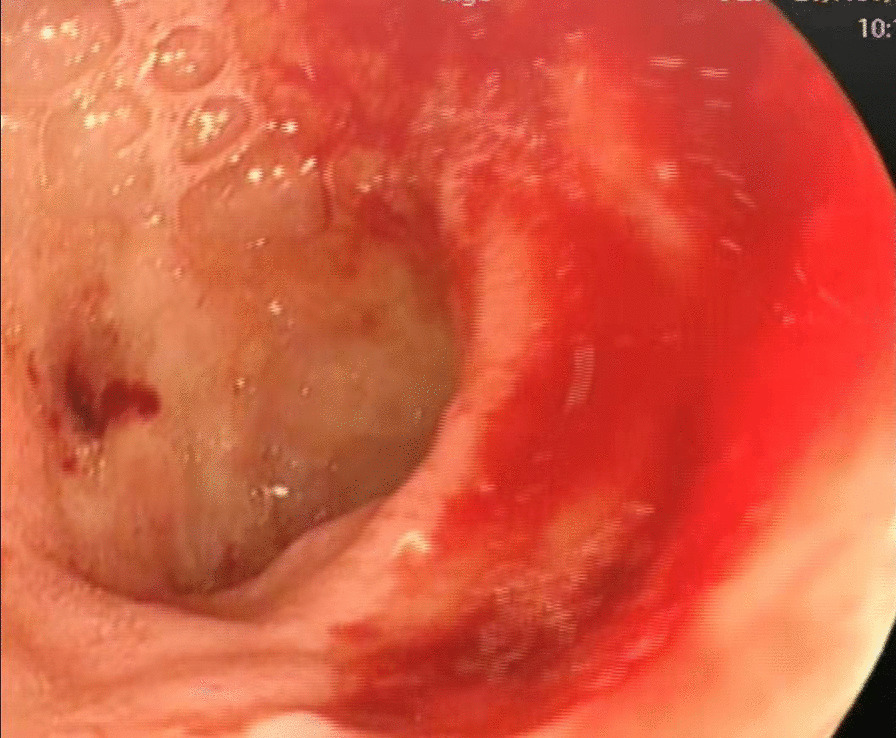
First endoscopy showing a 1.0 × 1.0 cm lesion with ulceration and active bleeding in the duodenal bulb. No active bleeding was observed after multipoint injection with solution consisting of L-HS-E

**Fig. 2:**
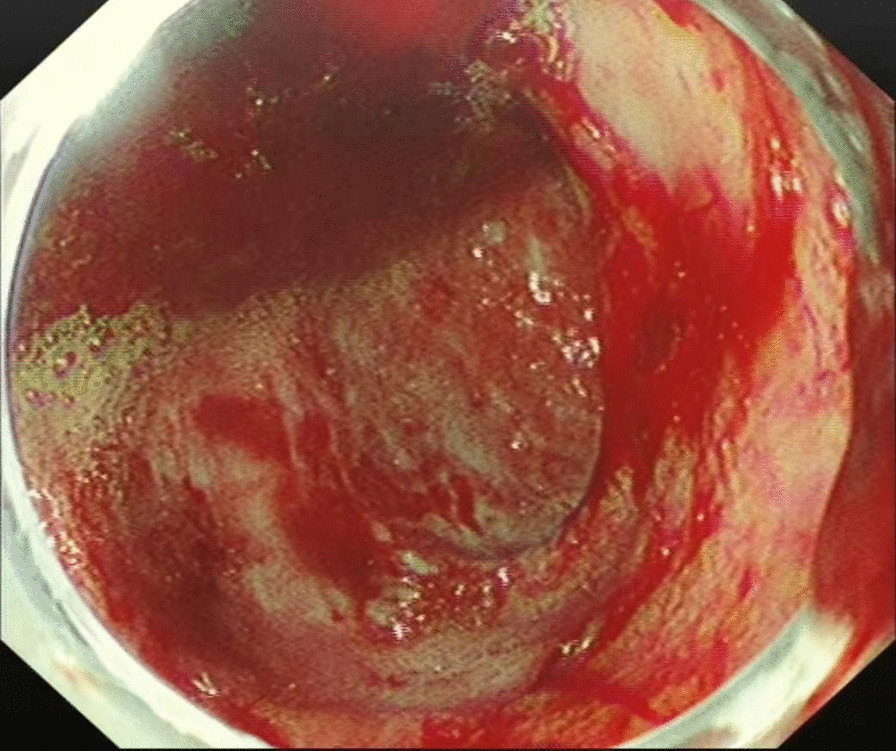
Second endoscopy showing a slightly depressed centre with active bleeding in the posterior wall of the duodenal bulb. L-HS-E was injected into 5 points, with 1–1.5 mL per point

**Fig. 3:**
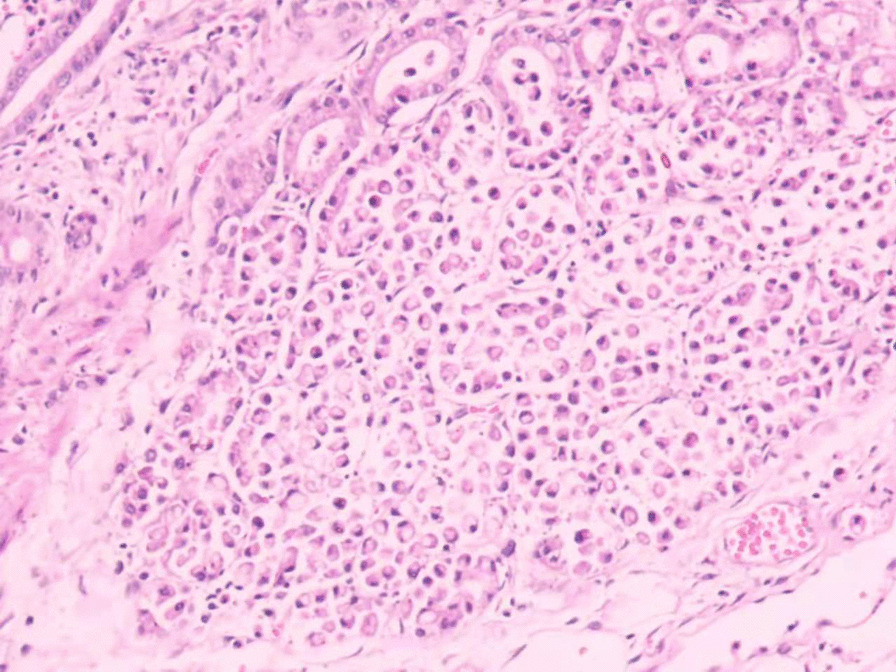
Pathological findings showing proliferation of SRCs with vacuolated foamy cytoplasm and displaced ovoid nuclei, resembling xanthoma cells (hematoxylin–eosin staining × 10)

**Fig. 4:**
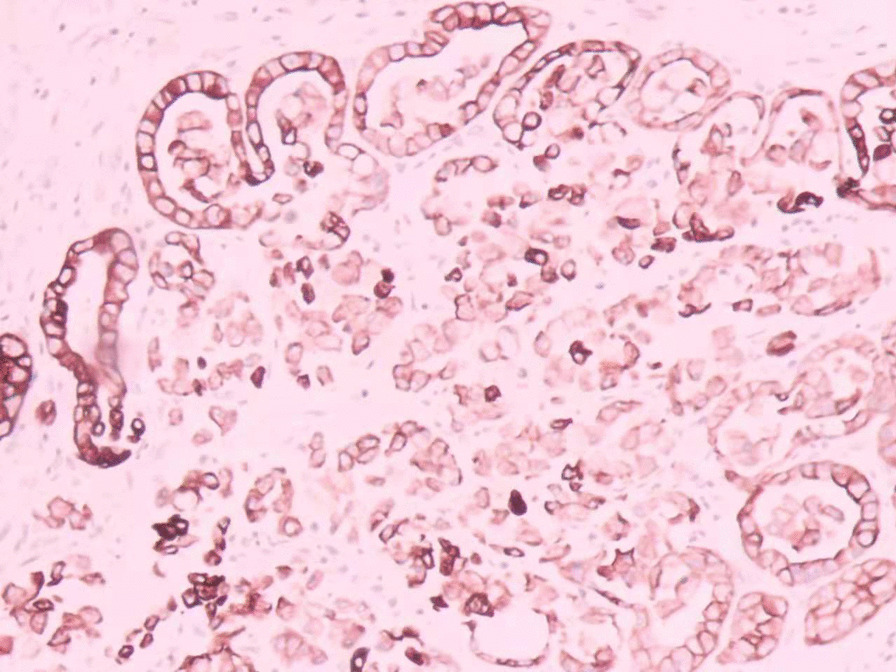
Positive staining of the AE1/AE3 immunohistochemical makers

**Fig. 5:**
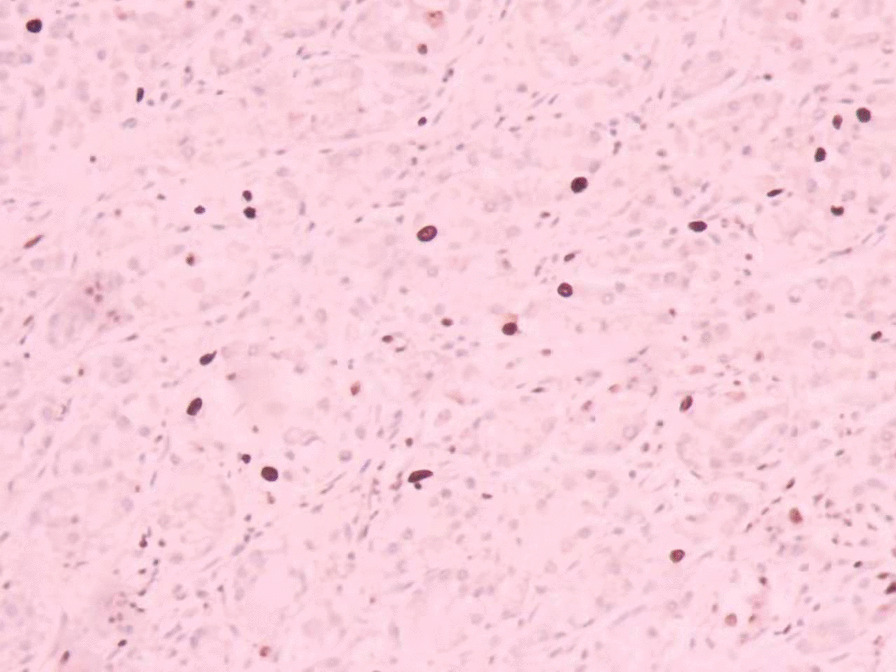
Immunohistochemistry showing a Ki-67 labelling index of approximately 10%

The patient was tachycardic, but vital signs were otherwise normal. Physical examination revealed tenderness in the epigastric region and a pale appearance, but was otherwise unrevealing. Laboratory values indicated anemia (hemoglobin of 94 g/L) and renal dysfunction (blood urea of 25.4 mmol/L and creatinine of 352 μmol/L). Tumor makers, such as carbohydrate antigen 19–9 (CA19-9), alpha-fetoprotein (AFP), carcinoembryonic antigen (CEA) and carbohydrate antigen 12–5 (CA12-5) were normal. Computed tomography (CT) and abdominal ultrasound showed no abnormalities. During hospitalization, the patient received two endoscopic hemostasis procedures and one interventional embolization. The first endoscopy revealed a 1.0 × 1.0 cm lesion with ulceration and active bleeding in the duodenal bulb. No active bleeding was observed after multipoint injection with a solution consisting of lidocaine, hypertonic saline and epinephrine (L-HS-E) (Fig. 1). Four days later, however, the patient had a feeling of epigastric discomfort. To decompress the stomach, drainage was performed with a nasogastric tube, and blood was observed. After discussion, an interventional embolization was performed but no contrast extravasation was observed in the gastroduodenal artery (GDA). According to the endoscopy results, GDA was embolized, but hemostatsis was not achieved. The patient then received a second endoscopy, which revealed a slightly depressed centre with active bleeding in the posterior wall of the duodenal bulb (Fig. 2). L-HS-E was injected into 5 points, with 1–1.5 mL per point. Unfortunately, adequate hemostasis was still not achieved. Persistent bleeding was observed in the nasogastric tube, and an increased heart rate appeared, indicating hemorrhagic shock. Considering that the patient was critical, distal subtotal gastrectomy, which included the duodenal bulb was performed to achieve adequate hemostasis. Duodenal bulb swelling with hemorrhage was found during the operation, and erosions with edema in the mucosal was significant. Postoperative histologic examination identified a 3 × 2.5 cm tumor consisting of proliferation of SRCs with vacuolated foamy cytoplasm and displaced ovoid nuclei, resembling xanthoma cells (Fig. 3). The tumor was limited to the mucosa and submucosal duodenal gland (pT1). No lymphovascular invasion, pancreatic invasion or metastasis was observed in 6 (4 in the lesser curvature and 2 in the greater curvature) resected lymph nodes (pN0). The proximal margin was negative for SRCs, but the distal margin was positive for SRCs. Immunohistochemical staining demonstrated a positive reaction for cytokeratin (AE1/AE3) in the carcinoma cells (Fig. 4), the Ki-67 labelling index was approximately 10% (Fig. 5). The p53, CerbB-2, CD68, CD56, Syn and CgA markers were negative.

After the operation, the patient still suffered from persistent gastrointestinal bleeding, and his family declined reoperation. After blood transfusion, melena and blood in the nasogastric tube gradually decreased, and enteral nutrition was provided. Within two weeks after the operation, extubation was attempted several times but failed. Lung CT revealed the presence of an air bronchogram, indicating atelectasis. Therefore, a fibreoptic bronchoscope was used to aspirate secretions. However, the patient’s respiratory function continued to worsen. Finally, tracheotomy was performed. Later, the patient’s hospital course was complicated by ileus. Moreover, signs of peritonitis appeared, and intra-abdominal infection (IAI) originating in the gastrointestinal tract was considered. Antibiotic treatment and other symptomatic therapies were given, but the symptoms did not improve. As time went by, the patient also suffered from an inflammatory process and gradually had a fever. The situation worsened, and a jejunal nutrient tube was placed instead of enteral nutrition. The patient died one month postoperatively.

## Discussion and conclusions

PDCs are rare tumors and are mainly located in the third and fourth portions of the duodenum (45%), followed by the second part of the duodenum (40%) and the duodenal bulb (15%) [6]. Abdominal pain, intestinal obstruction and bleeding are the most common symptoms of adenocarcinoma of the small bowel [7]. However, many patients have nonspecific cancer symptoms [2]. A literature review has reported that the diagnosis of PDCs may be difficult due to the following reasons: symptoms are either mild or nonspecific; tumor markers may be normal or only slightly elevated; and the sensitivity of biopsies is low [5]. Therefore, PDC is often misdiagnosed or missed in the early stage, and it is not diagnosed until symptoms, such as obstruction and jaundice, appear in the late stage [8]. In our case, the patient presented with repeated gastrointestinal bleeding accompanied by occasional epigastric discomfort. Ulceration with hemorrhage was considered previously, but no attention was paid seriously.

The development of advanced endoscopic and imaging methods, as well as an accurate histological examination, allows a better preoperative characterization of duodenal cancer. At present, the main clinical diagnostic methods for duodenal cancer include endoscopy, ultrasonography, magnetic resonance imaging (MRI) and enhanced CT [9]. Endoscopy is the diagnostic modality of choice for the evaluation of PDC, which allows simultaneous visualization and biopsy, with a diagnostic rate of 92.31% [10, 11]. Ultrasonography includes endoscopic ultrasound (EUS) and abdominal ultrasound. EUS can be used to evaluate local extension or lymphadenopathy simultaneously when endoscopy is performed. Abdominal ultrasound can find the dilation of the bile duct and pancreatic duct. However, it is easily affected by abdominal gaseous distension, leading to limitations in clinical application. Enhanced CT and MRI are widely used in assessing tumor depth invasion (T) and regional lymph node invasion (N), which play an important role in assessing the involvement of nearby structures, determining resectability and planning surgery [10, 12]. In cases without a confirmed diagnosis, sensitive but nonspecific radiographic features suggestive of malignancy include an exophytic or intramural mass, central necrosis and ulceration. In addition, for tumors with rich blood supply and hemorrhage, the technique of selective digital subtraction arteriography (DSA) can observe vessel infiltration of the tumor, which is helpful for diagnosis and surgery selection [8].

Surgery is the only radical treatment for SRCC in the duodenum. It has been reported that the 5-year survival rate after radical resection of PDC is approximately 37%-55% [13, 14]. However, there is no clinical data on the 5-year survival rate after radical resection of SRCC, which may be due to the small sample size of cases, indicating that further study is required in the future. Surgical resection is accomplished via four major procedures, including transduodenal excision (transduodenal submucosal excision), local full-thickness resection (wedge resection), pancreas-sparing segmental duodenectomy (PSD), and pancreaticoduodenectomy (PD) [15]. PD has been considered the preferred surgical option for PDC [7, 16, 17]. Cancer can be treated by surgery simultaneously with lymphadenectomy. However, PD is complicated with a high risk of operation and postoperative complications such as biliary fistula and pancreatic fistula. The other three procedures are so-called “limited resections” and are generally utilized for selected tumors not amenable to endoscopic resection that have no or negligible risk of nodal metastasis. Therefore, it may be acceptable that the selection of resection method is determined on the basis of tumor size and histopathological findings of a lymph node using an intraoperative frozen section. For those who are in the late stage of cancer, a palliative operation can be performed to relieve the obstruction of the gastrointestinal or biliary tract, improving the quality of life. Cholangiojejunostomy is often performed together with Roux-en-Y gastrojejunostomy or duodenojejunostomy [18]. Recently, Okamoto T et al. reported that endoscopic resection may be a viable alternative for early SRCC limited to the mucosal layer, thereby providing a new option [5]. In our case, emergency surgery was performed with the main purpose of hemostasis. Only the lesion with hemorrhage was removed during the operation instead of radical resection.

Histologically, there are several theories about the origin of SRCs. One theory suggests that SRCs originate in ectopic gastric mucosa found in the duodenum, while another theory suggests that SRCC arises from gastric type metaplastic epithelium [19–21]. In SRCC, the accumulation of mucins such as MUC5AC and MUC2, results in large vacuoles, which may play a role in carcinogenesis [22]. Unfortunately, our hospital could not evaluate mucins, such as MUC5AC or MUC2, due to technical limitations.

The prognosis of SRCC is poor, which may be due, in part, to its more advanced stage. Reports have shown that over 80% of SRCC diagnoses present with advanced disease regardless of the site of origin [19, 20]. In a review of 4995 patients with small bowel adenocarcinoma reported to the National Cancer Database between 1985 and 1995, the overall 5-year survival was 30.5%, and the median survival was 19.7 months; the 5-year survival rates for stage I, II, III and IV were 65%, 48%, 35% and 4%, respectively [23]. Lymph node involvement, curative resection of the tumor and localization of the tumor have an impact on patient survival [24–27]. In our case, the surgical margin was positive, indicating the poor prognosis of the patient.

Whereas SRCC is thought to be less chemosensitive than non-SRCC, it may have a specific sensitivity profile and be more sensitive to taxane-based chemotherapy or antiangiogenics [28–30]. Bang YJ et al. reported that immunotherapy should be tested in SRCC as PDL1 is overexpressed in approximately 23% of cases of SRCC, indicating that an anti-PDL1 monoclonal antibody may be a promising treatment [31]. However, reports have also shown that oncological treatment, such as adjuvant chemoradiotherapy, does not improve survival [13, 25, 32]. Therefore, more prospective trials are needed in the future.

In the present case, the tumor was considered to be primary duodenal bulb cancer due to several reasons. (1) Duodenal ulcer was indicated by endoscopy 2 months ago, and no other lesions were found in the stomach. The patient received a proton pump inhibitor (PPI), but his symptoms did not improve. (2) According to the pathology, the tumor was limited to the mucosal layer and submucosal duodenal gland. The proximal margin was negative, while the distal margin was positive. (3) SRCC was not detected in the stomach histopathologically, and no mass was found elsewhere during the operation. Because of the extensive vascular network that increases the risk of bleeding and the thin, deep muscle layer leading to high perforation rates, duodenal biopsy is not routine, and an experienced endoscopist is critical when performing endoscopic evaluation [33]. For patients whom are diagnosed with ulcers, if symptoms are not improved after regular treatment or the lesion is not healing well when re-examined by endoscopy, pathological biopsy is recommended based on the present case. Multiple samplings should be performed if necessary. Clinically, endoscopy is recommended for patients with dyspepsia aged over 50 or those with alarm features, such as family history of upper-gastrointestinal malignancy, unintended weight loss, gastrointestinal bleeding, iron deficiency anemia, progressive dysphagia, odynophagia, persistent vomiting, palpable mass, lymphadenopathy or jaundice. If there is a clinical suspicion of malignancy, even in the absence of alarm features, endoscopy should also be considered [34].

In conclusion, we report a rare case of SRCC in the duodenal bulb. The diagnosis of PDCs is difficult due to mild and nonspecific presenting symptoms, normal or only minimally elevated tumor markers, and low sensitivity of biopsies. For patients who can receive radical tumor resection, PD should be considered as the first choice. The prognosis of PDC is potentially improved through early diagnosis and radical surgical resection.

## Data Availability

All patient data and medical images can be found in the database of the Information Office of Dongyang People’s Hospital.

## Abbreviations

SRCC: Signet-ring cell carcinoma
PDC: Primary duodenal cancer
PD: Pancreaticoduodenectomy
CA19-9: Carbohydrate antigen 19–9
AFP: Alpha-fetoprotein (AFP)
CEA: Carcinoembryonic antigen
CA12-5: Carbohydrate antigen 12–5
CT: Computed tomography
L-HS-E: Lidocaine, hypertonic saline and epinephrine solution
GDA: Gastroduodenal artery
IAI: Intra-abdominal infection
MRI: Magnetic resonance imaging
DSA: Digital subtraction arteriography
PSD: Pancreas-sparing segmental duodenectomy
PPI: Proton pump inhibitor
